# Administration of sodium hydrosulfide reduces remote organ injury by an anti-oxidant mechanism in a rat model of varicocele

**DOI:** 10.22038/IJBMS.2019.39727.9412

**Published:** 2020-02

**Authors:** Keivan Lorian, Mehri Kadkhodaee, Farzaneh Kianian, Arash Abdi, Behjat Seifi

**Affiliations:** 1 Department of Physiology, School of Medicine, Tehran University of Medical Sciences, Tehran, Iran

**Keywords:** Infertility, NaHS, Oxidative stress, Rat, Remote organs, Varicocele

## Abstract

**Objective(s):**

Infertility in varicocele may have an adverse outcome on the future life of an infertile male. This study was designed to investigate whether varicocele affects remote organs, including the kidney, liver, and brain. We have also evaluated the protective effects of NaHS administration on the structure and function of these organs.

**Materials and Methods:**

Thirty-six rats were randomly assigned to 3 experimental groups: 1) Sham, 2) Varicocele, and 3) Varicocele + sodium hydrosulfide. Varicocele was induced via partial ligation of the left renal veins. Animals in the Varicocele + sodium hydrogen sulfide group received 30 µmol/l NaHS in drinking water for 56 days. On the 57th day of the treatment, blood samples, as well as kidney, liver, and brain tissues, were collected to assess kidney and liver functions, measurement of oxidative stress markers, and histological changes. For evaluation of sperm parameters caudal epididymis was used. The behavioral tests were performed to evaluate the animal’s anxiety-related behaviors.

**Results:**

Varicocele caused significant decrease in sperm parameters (motility and viability) and superoxide dismutase activity in the kidney, liver, and brain tissue. Anxiety-related parameters decreased in varicocele. Moreover, varicocele resulted in a significant increase in malondialdehyde levels in the kidney, liver and brain tissue, and liver function enzymes. Varicocele did not alter kidney function parameters. The administration of NaHS improves the above parameters.

**Conclusion:**

This study showed that notice to remote organs such as the liver and brain beside reproductive organs in varicocele is important. The administration of NaHS improved remote organ injury in varicocele via its anti-oxidant mechanism.

## Introduction

Male infertility refers to male inability to fertilize a healthy female, which affects approximately 7% of the male population (1). It has a complex etiology, which includes poor semen quality, varicocele, endocrine disorders and other factors (2). Infertile men who diagnose their infertility are more vulnerable to incidents of chronic medical conditions. They have higher rates of ischemic heart disease, diabetes, depression, and other conditions, which may have adverse results on their future life (3). In addition, infertile individuals have a higher risk of autoimmune diseases such as Graves’ disease and multiple sclerosis. These show the relationship between fertility and the immune system (4). Mental health problems like depression and anxiety are other disorders observed in infertile men that state the association between infertility and psychological conditions (2). Early understanding and diagnosing harmful outcomes of infertility on the health of other organs in infertile males and proper intervention is important. The prevalence and other essential details about male factor infertility are known, but more investigation is needed to understand the adverse results of these factors in later life. There is limited data on the future health of infertile men because the ability to find outcomes of infertility is limited (3), thus using experimental varicocele as a male infertility model could be an appropriate way to explore these outcomes. 

Varicocele is defined as abnormal dilatation and tortuosity of the testicular pampiniform plexus vein, which drains the testis (5). Varicocele, which affects approximately 15% of the male population, is one of the common causes of male infertility, which is also associated with a decrease in sperm quality (6). Studies on fertile and infertile men showed that serum malondialdehyde (MDA) and total antioxidant capacity (TAC) are related to sperm count and motility (7). Elevated levels of reactive oxygen species (ROS) and reduced levels of antioxidants were detected in both semen and blood in patients with varicocele (8). Lipid peroxide, the free radical peroxidation of lipids, is an important element in local injury, which can damage enzyme activity and membrane receptors can also affect the other remote organs (9). 

In clinical research, NaHS has been used widely to evaluate the biological effects of H_2_S (10). This chemical compound had various protective effects, such as antioxidative and anti-inflammatory effects. Azizi *et al.* reported the protective effects of NaHS against oxidative stress in ischemia-reperfusion to induce acute kidney injury in the rat model (11). NaHS administration improved renal function in cirrhotic rats (12). Moreover, long term administration of NaHS had protective effects on chronic kidney disease via antioxidant, anti-inflammatory, and anti-apoptotic properties (13). 

The interactions between varicocele-induced testis injury due to squeezing the left renal vein and distant organs remain unclear. Therefore, the current study was designed to evaluate remote organs, including the kidney, liver, and brain in varicocele rats, and to assess the protective effects of NaHS in this disorder. 

## Materials and Methods

***Animal procedure***

In this study, thirty-six male Wistar rats weighing 200–250 g were used. Animals were kept in the animal laboratory in a controlled environment, a cycle of 12 hr dark–light and 22±2 ^°^C temperature, with free access to food and water. All experiments were approved by the Ethics Committee of Tehran University of Medical Sciences. Animals were randomly assigned into 3 groups of 12 each: Sham, Varicocele, and Varicocele + NaHS.

***Varicocele induction***

Induction of the experimental varicocele as the partial ligation of the left renal vein was performed by using the Turner’s method (14). Each animal was anesthetized with an intraperitoneal injection of ketamine hydrochloride 100 mg/kg and xylazine 10 mg/kg. The incision of abdominal midline was made by surgical scissors, then contents of the abdomen were shifted to the right side of the body by moisturizing gauze; the left renal vein, inferior vena cava, left spermatic vein, and insertion of left spermatic vein into the left renal vein were then recognized. Surroundings of the left renal vein were cleaned from fat and connective tissues in order to create a tunnel beneath the vein, medial to the insertion of the left spermatic vein. A 4-0 silk suture was placed loosely around the left renal vein and tied over a needle that had a 0.7 mm diameter and was located parallel to the left renal vein; then, the needle was slowly removed to allow the vein to expand to the limit of the ligation. Then the contents of the abdomen were replaced, and the incision was closed in two layers by 4-0 silk suture. The sham group underwent the same procedure, but the partial ligation of the left renal vein was not performed. This type of varicocele induction had no harmful effect on the left kidney. 

***Drug***

After one week of recovery, all varicocele rats were equally randomized to receive drinking water with or without NaHS 30 µmol/l daily for 56 days. The treatment period was chosen according to spermatogenesis in rats (15), and the dose of NaHS was chosen based on its protective effects on the 5/6 nephrectomized rats (13). 

***Sample collection and preparation***

On the 57th day of treatment, the rats were again anesthetized, and blood, liver, kidney, and brain tissues were collected. Blood samples were obtained from inferior vena cava, centrifuged at 4000 g for 10 min at 4 °C. Plasma was obtained and stored at –70 °C until use. After blood sample collection, the portions of liver, kidney, and brain tissues were frozen for the measurement of oxidative stress markers. The other parts of the liver, kidney, and testis tissues were kept in 10% formalin for H&E staining. The left epididymis was dissected and washed with saline; the caudal part was used for the evaluation of sperm parameters. 

***Sperm collection***

For sperm preparation, the sperm washing media was pre-warmed at 37 ^°^C in an incubator. Next, the caudal part of epididymis was minced with surgical scissors and incubated in 5 ml washing sperm media for 15 min (16).

***Evaluation of the sperm parameters***

*Sperm motility*

Ten microliters of sperm suspension were placed on pre-warmed slides, and then the motility of 200 sperms at ten microscopic fields was assessed under a 400×magnification phase-contrast microscope (Olympus, Germany) according to WHO 2010 recommendations. The percentages of progressive motile sperm (PMS), non-progressive motile sperm (NPMS), and non-motile sperm (NMS) were recorded (17).

*Sperm viability*

 To assess the sperm viability, eosin-nigrosin staining was used. Briefly, eosin (1%, Merck, Germany) and nigrosin (10%, Merck, Germany) were prepared in distilled water, and two volumes of 1% eosin were mixed with one volume of sperm suspension. An equal volume of 10% nigrosin was added After 30 sec. Thin smears were prepared, and the viability of 100 sperms was assessed under a 100×magnification light microscope (Olympus, Germany). The dead sperms stained pink while viable sperms remained colorless. The percentage of viable sperms was recorded (18). 

***Biochemical assay***

The renal parameters, levels of blood urea nitrogen (BUN), plasma creatinine (Cr), and urine protein concentration were measured by an automatic analyzer (Hitachi, Ltd., Tokyo, Japan). 

Liver function indices, alanine aminotransferase (ALT), and aspartate aminotransferase (AST) were evaluated by colorimetric methods using commercially available kits.

***Measurement of MDA & SOD as the oxidative stress markers of kidney, liver, and brain tissues***

MDA level was measured based on the Esterbauer and Cheeseman methods. MDA reacts with thiobarbituric acid to produce a pink pigment with maximum absorption at 532 nm (19).

Superoxide dismutase (SOD) activity was measured based on the Paoletti and Mocali methods. Briefly, in the presence of ethylene diamine tetraacetic acid, superoxide anions are produced by manganese (II) chloride and mercaptoethanol. NADPH oxidation is related to the accessibility of superoxide anions, which is measured at 340 nm (20).

***Behavioral testing***

*Elevated plus-maze test*

This test was performed to assess anxiety-related behavior in the animals (21). The apparatus is made up of four arms, which was 35 cm long and 5 cm wide; the two open arms had 0.5-cm-high edges, and two closed arms had 15-cm-high dark walls. The height of the maze from the floor was 50 cm.

After 56 days of treatment, before sampling, all rats were placed individually in the center of the maze facing an open arm. The number of entries and the spending time in the open or closed arms were recorded during the next 5 min. The arms were cleaned with 70% ethanol between sessions. 

The percentages of open arm entries (100×number of open arm entries/total entries into all arms) (%OAE) and spending time in open arms (100× time on open arms/total time spent in all arms) (%OAT) were calculated for each animal. Increased open arm activity (entry and time) demonstrates reduced anxiety-related behaviors. The total entries (sum of the number of entries into open and closed arms) were evaluated as a locomotor activity indicator.

*Light/dark box*

The light/dark box was also used to assess anxiety-related behaviors (21). The apparatus consisted of a Plexiglas box with two equal chambers (30 × 40 × 40 cm); one had white walls and floor, which was illuminated by a 60-watt light from above, while the other chamber was black and had a lid with no illumination. Between the two chambers was an 8 cm × 8 cm square hole that the rats could use to move between them. Each animal was placed in the light chamber of the box, and the time spent in the light side was recorded over the next 5 min. The increased time in the light chamber shows decreased anxiety-related behaviors. After each test session, the apparatus was cleaned.

***Histological procedure***

Liver, kidney, and testis tissues were fixed in 10% formalin buffer, then dehydrated and embedded in paraffin. Hepatic, renal, and testicular sections of four micrometers were stained with hematoxylin-eosin (H&E).

Kidney tissues were evaluated for the presence of tubular injury as cast formation, congestion, and tubule dilation. 

Liver tissues were evaluated for sinusoidal congestion and disintegration of the cellular cords.

Testis tissues were evaluated for lumina, epithelium, and interstitium changes of seminiferous tubules.

***Statistical analysis***

All collected Data are expressed as mean±SEM. One-way analysis of variance followed by Tukey’s *post hoc* test was used to compare the different groups. *P*<0.05 was considered statistically significant**.**

## Results

***Varicocele assessment***

**Figure 1:**
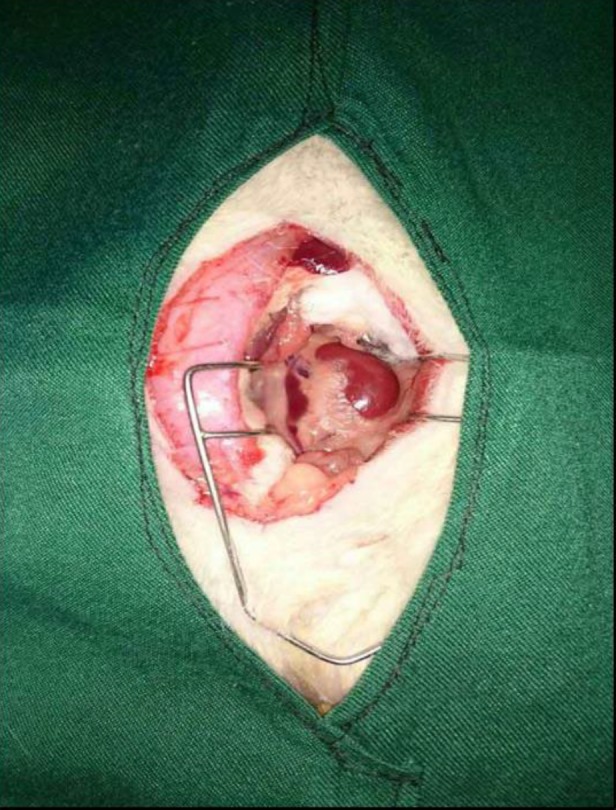
Dilation of the left spermatic vein 9 weeks after partial ligation of the left renal vein

Dilation and swelling, which were observed in the left spermatic vein after nine weeks, confirmed that varicocele had been induced successfully in the animals of this study (Figure 1).

***Sperm parameters during varicocele and after NaHS administration***

**Figure 2:**
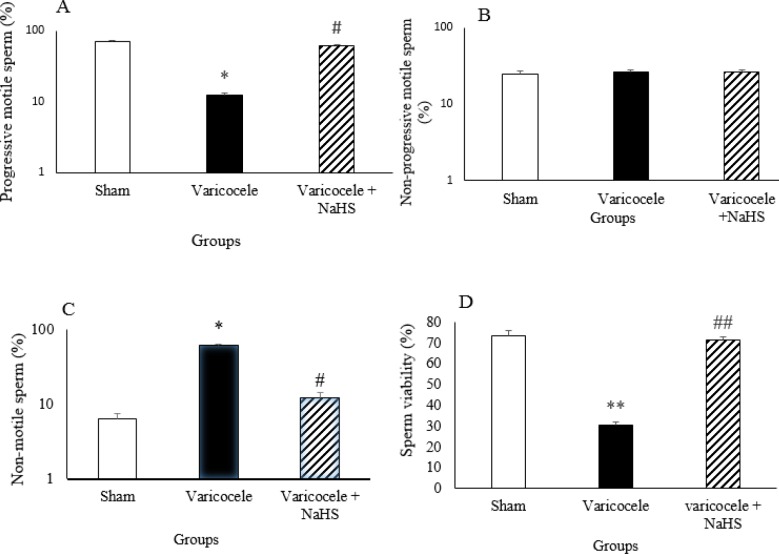
Sperm motility (A, B, and C) and sperm viability (D) in various groups of experiment. Data are presented as mean+SEM. *P<0.05 significant differences vs Sham group. #*P*<0.05 significant differences vs Varicocele group. **P<0.01 significant differences vs Sham group. ##*P*<0.01 significant differences vs Varicocele group

Varicocele resulted in a significant decrease in the percentage of PMS and significant increase in the percentage of NMS compared with the sham group but did not alter the percentage of NPMS. (Figures 2 A, B, and C, *P*<0.05). NaHS administration increased the percentage of PMS and decreased NMS compared with the varicocele group. The percentage of NPMS was not altered.

Varicocele caused a significant decrease in the percentage of viable sperms compared with the sham group. (Figure 2 D, *P*<0.01). NaHS administration increased the percentage of viable sperms compared with the varicocele group.

***Renal parameters during varicocele and after NaHS administration***

Varicocele did not alter renal parameters (BUN, plasma creatinine, and urine protein) compared with the sham group (Table 1). Not surprisingly. NaHS administration did not alter the above parameters compared with the sham group.

***Liver function indices during varicocele and after NaHS administration***

**Figure 3:**
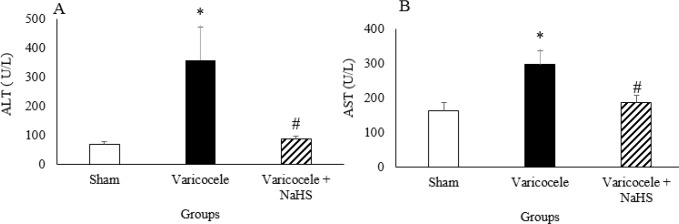
Liver function indices. Alanine aminotransferase (ALT) (A) and aspartate aminotransferase (AST) (B) in different groups of experiment. Data are presented as mean+SEM. *P<0.05 significant differences vs Sham group. #*P*<0.05 significant differences vs Varicocele group

Varicocele caused significant increase in liver function indices (blood AST and ALT levels) compared with the sham group. (Figures 3 A and B, *P*<0.05). NaHS administration decreased these indices compared with the varicocele group.

***Oxidative stress markers during varicocele and after NaHS administration***

**Figure 4.**
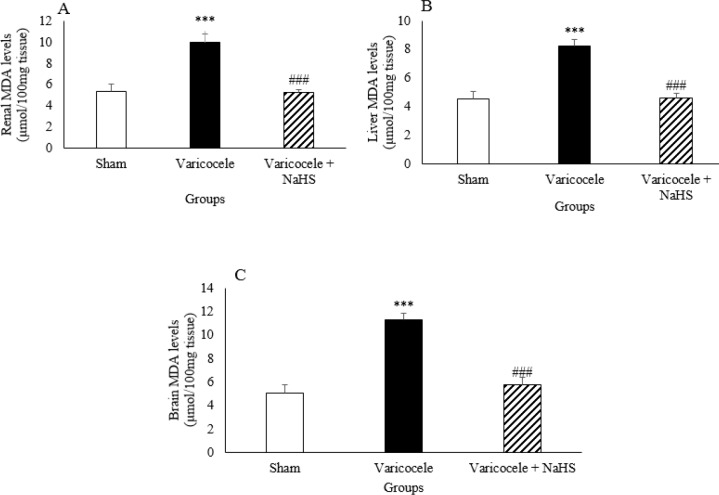
MDA levels in kidney (A), liver (B), and brain (C) in different groups of experiment. Data are presented as mean+SEM. *** *P*<0.001 significant differences vs Sham group. ### *P*<0.001 significant differences vs Varicocele group

Varicocele resulted in significant increase in the kidney, liver, and brain tissue MDA levels compared with the sham group (Figures 4 A, B, and C, *P*<0.001). NaHS administration decreased MDA levels in these organs compared with the varicocele group.

**Figure 5:**
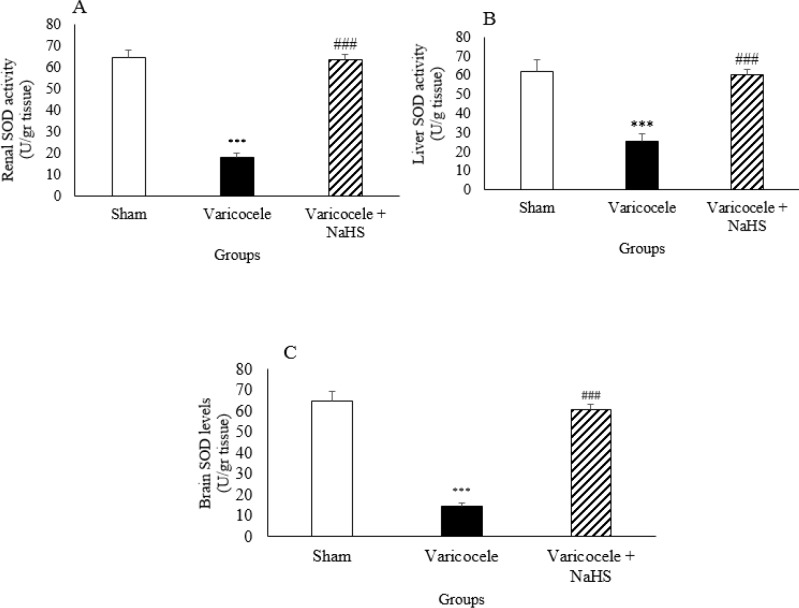
SOD activity in the kidney (A), liver (B), and brain (C) in different groups of experiment. Data are presented as mean+SEM. *** *P*<0.001 significant differences vs Sham group. ### *P*<0.001 significant differences vs Varicocele group

Varicocele caused significant decrease in kidney, liver, and brain tissue SOD activity compared with the sham group (Figures 5 A, B, and C, *P*<0.001). NaHS administration increased SOD activity in these organs compared with the varicocele group.

***Behavioral tests during varicocele and after NaHS administration***

**Figure 6:**
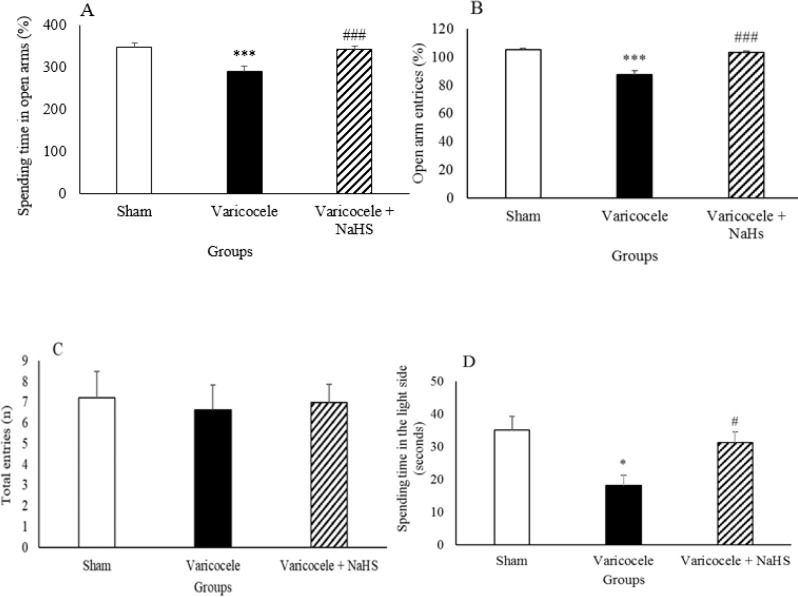
Spending times in open arms (A), changes in the percentages of open arm entries (B), total entries (C), and spending time in the light side (D) in different groups of experiment. Data are presented as mean+SEM. *** *P*<0.001 significant differences vs Sham group. ### *P*<0.001 significant differences vs Varicocele group. * *P*<0.05 significant differences vs Sham group. #*P*<0.05 significant differences vs Varicocele group

There was a significant decrease in the percentage of time spent in the open arms in the varicocele group compared with the sham group (Figure 6 A, *P*<0.001). Administration of NaHS significantly increased the percentage the time spent in the open arms in comparison with the varicocele group.

There was a significant decrease in the percentage of open arm entries in the varicocele group compared with the sham group (Figure 6 B, *P*<0.001). NaHS significantly increased the percentage of open arm entries in comparison to the varicocele group.

There were no significant differences in total entries between animals in sham, varicocele, and varicocele + NaHS groups.

There was a significant decrease in the time spent in the light side in the varicocele group compared with the sham group (Figure 6 D, *P*<0.005). The administration of NaHS significantly increased the time spent in the light side in comparison with the varicocele group.

***Histological changes during varicocele and after NaHS administration***

**Figure 7:**
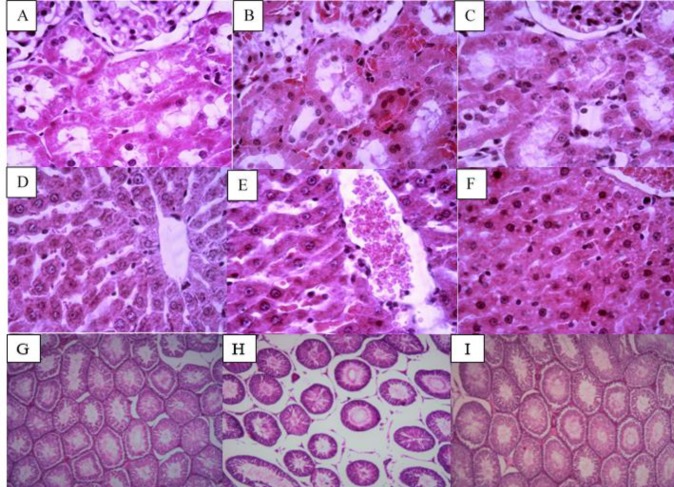
Changes in renal, liver, and testis histopathology in different groups of experiment. Kidney tissues: (A: Sham, B: Varicocele, C: Varicocele + NaHS). Liver tissues: (D: Sham, E: Varicocele, F: Varicocele + NaHS Testis tissues: G: Sham, H: Varicocele, I: Varicocele + NaHS.) Magnification ×400

The kidney tissues in the sham group showed normal histology. There were mild tissue changes in the varicocele group, demonstrating as mild interstitial and vascular congestion, tubular dilation, and mild cast formation. No significant changes were observed with the NaHS treatment (Figures 7 A, B, and C).

For liver tissues, the sham group had no evidence of damage. In the varicocele group, there was some leucocyte infiltration, sinusoidal and other vascular congestion was present, and a mild decrease in cellular cord arrangement was observed. No significant changes were observed with the NaHS treatment (Figures 7 D, E, and F). 

The testis tissue in the sham group showed almost normal histology. Wider lumina of seminiferous tubules were present. Some areas in the interstitium were evidently wider than normal. Administration of NaHS showed less structural changes compared with the varicocele group (Figures 7 G, H, and I).

**Table 1:** Renal parameters in various groups. Data are presented as mean+SEM

Parameters	Parameters		Varicocele + NaHS
	Sham	Varicocele	
BUN (mg/dl)	26.40±2.46	29.33±3.28	24.42±1.27
Plasma Cr (mg/dl)	0.80±0.04	0.85±0.07	0.77±0.03
Urine protein (mg/dl)	38±9.1	46.20±4.56	32.73±3.04

## Discussion

In the present study, varicocele induced liver damages and caused anxiety in rats, although kidney function was normal. The results represented oxidative stress in the liver, brain, and kidneys. Long-term administration of NaHS improved damages in these remote organs besides sperm parameters. Concerning this data, it can be concluded that detrimental consequences of infertility for the health of the other organs affect the future life of the infertile individuals. Determining chronic medical conditions in males with infertility is important for the reduction of probable complications of these diseases. 

Liver parameters such as ALT and AST were increased by varicocele besides changes in hepatic oxidative stress markers. The liver is the main organ attacked by ROS. When an excess amount of ROS as a result of varicocele accumulated in the liver, impaired hemostasis may play a major role in liver damage (22). Melgarejo *et al.* showed that in infertile men, both MDA and TAC increased in the blood similar to seminal fluid, comparable to another study that reported increased ROS in patients within both blood and seminal fluid while the antioxidants were decreased in both (7, 8). Liver dysfunction leads to the production of end products, AGEPs (advanced glycation end-products), and facilitates the production of free radicals through impairment of the endogenous scavengers such as superoxide dismutase (23, 24). NaHS treatment improved liver function and oxidative stress as a remote organ in the present study. It was reported that in cirrhotic rats NaHS administration ameliorated liver function and improved renal impairment (12). 

Although kidney function was not affected by varicocele, oxidative stress markers showed significant alterations. In nutcracker syndrome the left renal vein (LRV) is squeezed between the aorta and the superior mesenteric artery (SMA). It can cause LRV pressure elevation, varicocele, and development of collateral veins (25). This syndrome is similar to our experimental varicocele model, which is associated with the elevation of the left renal vein pressure, and normal kidney function confirms our precise method without direct damage to the neighboring kidney. Similar to our study, Takemura *et al.* (26) reported no changes in the renal biopsy of patients with this syndrome. Ko *et al.* (27) observed no fibrotic changes in the left kidney during varicocele. However, in the current study, renal oxidative stress was observed and NaHS treatment improved this disturbance. This finding may show us kidneys are susceptible to damage secondary to varicocele, and considering their health is important and vital.

Behavioral tests (elevated plus-maze and light/dark box) as indicative factors of brain health were changed in the varicocele rat model. Studies demonstrate that infertility causes anxiety and depression in men. A study found that infertile men were susceptible to psychological distress (1). Ketabchi reported that patients with varicocele were suffering from mental disorders, which may interfere with their treatment (28). Reduction of testosterone levels in patients with varicocele and in animals was reported (29). It was shown men who had low levels of testosterone were also suffering from anxiety and depression disorders (30). However, in our study we did not measure testosterone, oxidative stress observed in the brain may be due to lower testosterone and cause behavioral changes in the varicocele rat model. NaHS treatment in our study improved anxiety in the varicocele model by reducing oxidative stress. Therefore, diagnosis and treatment of male infertility is vital in order to prevent the development of later morbidity. 

## Conclusion

Taken together, harmful effects of varicocele on sperm parameters, which lead to infertility, may disturb remote organs such as the liver and brain via its principal pathophysiology mechanism: oxidative stress. Thus, notice to remote organs beside reproductive organs should be considered in varicocele-induced infertility. Treatment using NaHS provided a protective effect against sperm parameters and remote kidney, liver, and brain dysfunction through the attenuation of oxidative stress. 
